# Research led by people who use drugs: centering the expertise of lived experience

**DOI:** 10.1186/s13011-021-00406-6

**Published:** 2021-09-20

**Authors:** Zach R. Salazar, Louise Vincent, Mary C. Figgatt, Michael K. Gilbert, Nabarun Dasgupta

**Affiliations:** 1 North Carolina Survivors Union, 1116 Grove Street, Greensboro, NC 27403 USA; 2 Injury Prevention Research Center, University of North Carolina At Chapel Hill, Chapel Hill, NC USA; 3 Department of Epidemiology, Gillings School of Global Public Health, University of North Carolina At Chapel Hill, Chapel Hill, NC USA; 4 Independent Researcher, Portland, OR USA

**Keywords:** Harm reduction, People who use drugs, Community-led research, Syringe service programs, Drug user unions, Community driven research

## Abstract

**Background:**

Research collaborations between people who use drugs (PWUD) and researchers are largely underutilized, despite the long history of successful, community-led harm reduction interventions and growing health disparities experienced by PWUD. PWUD play a critical role in identifying emerging issues in the drug market, as well as associated health behaviors and outcomes. As such, PWUD are well positioned to meaningfully participate in all aspects of the research process, including population of research questions, conceptualization of study design, and contextualization of findings.

**Main body:**

We argue PWUD embody unparalleled and current insight to drug use behaviors, including understanding of novel synthetic drug bodies and the dynamics at play in the drug market; they also hold intimate and trusting relationships with other PWUD. This perfectly situates PWUD to collaborate with researchers in investigation of drug use behaviors and development of harm reduction interventions. While PWUD have a history of mistrust with the medical community, community-led harm reduction organizations have earned their trust and are uniquely poised to facilitate research projects. We offer the North Carolina Survivors Union as one such example, having successfully conducted a number of projects with reputable research institutions. We also detail the fallacy of meaningful engagement posed by traditional mechanisms of capturing community voice. As a counter, we detail the framework developed and implemented by the union in hopes it may serve as guidance for other community-led organizations. We also situate research as a mechanism to diversify the job opportunities available to PWUD and offer a real-time example of the integration of these principles into public policy and direct service provision.

**Conclusion:**

In order to effectively mitigate the risks posed by the fluid and volatile drug market, research collaborations must empower PWUD to play meaningful roles in the entirety of the research process. Historically, the most effective harm reduction interventions have been born of the innovation and heart possessed by PWUD; during the current overdose crisis, there is no reason to believe they will not continue to be.

## Background

Research collaborations between people who use drugs (PWUD) and researchers are underacknowledged, misinterpreted, and undervalued. The most effective evidence-based harm reduction interventions, such as needle-exchange [1], peer-delivered naloxone [2] and community-based drug checking [3], were designed and implemented by PWUD well before they had the backing of the greater public health community. PWUD play significant roles in identifying cross-disciplinary issues of import and contextualizing significance of findings. Therefore, collaborations with PWUD are necessary to produce effective and relevant research. Ultimately, we aim to argue the necessity of research collaborations with PWUD; detail the optimal positioning of harm reduction organizations in facilitating research projects; provide a framework for successfully conducting these partnerships; propose a potential career path for PWUD; and provide real-world application of the presented concepts.

## Community contextualization

PWUD are experiencing a fluid illicit drug market where changes to products and potency can be extremely volatile, posing a series of unpredictable, and potentially deadly, risks to the consumer. The recent increase in novel synthetic drug types bodes ill for public health and safety because new psychoactive substances (e.g., fentanyl analogs) are frequently unknown by law enforcement and undetected by conventional drug screens, with associated health risks unfamiliar to public health. Evidently, though, people who consume these substances know what they are, or what they are intended to imitate, including what they look like, the places where they are sold, how much they cost, who is using them, methods and techniques for using (e.g., routes of administration), and the social network dynamics that facilitate contacts between consumers (i.e., dyadic pairings) [4].

The knowledge inhabited by PWUD, and the trusting and intimate relationships they have with other PWUD and their social networks, begs for collaboration in all areas of research involving illicit drug use or the population of PWUD. PWUD are needed at all stages of the research process: from conceptualizing ideas and constructing research questions to interpreting study findings and helping develop effective responses.

## Situatedness of harm reduction organizations

PWUD are conventionally mistrusting and skeptical of medical professionals and social workers, and rightfully so, due to a long history of mistreatment and deeply ingrained stigma. Harm reduction organizations, such as syringe service programs (SSPs), and the workers who deliver services have secured the trust and respect of participants and have a unique opportunity to provide health care services and mobilize community involvement in research [5, 6].

When researchers view PWUD solely as research subjects and within a strict binary of researcher/researched—where PWUD are interpolated as either vectors of information or a means to recruit study participants—they fail to recognize (or communicate) how circumstantial complications can influence health outcomes and how the combined factors of agent, host, and environment contribute to disease transmission and/or acquisition of injury. By collaborating with people who actually perform the drug use behaviors under investigation in a genuine and transparent relationship, all components of the research process described above are enhanced. For example, the North Carolina Survivors Union (NCSU), a robust, drug-user union in central North Carolina, maintains strong professional relationships with researchers and academic institutions and welcomes collaboration. NCSU has served as a subcontractor on numerous NIH, FDA, and industry-funded research projects. The union has been involved in cutting edge research led by PWUD, including hepatitis testing and linkage-to-care, fentanyl test strip distribution [3], and community-based drug checking. Successful collaboration between NCSU and external research organizations has been driven by attainment of genuine “meaningful collaboration.”

Tokenistic attempts at involving PWUD in research (i.e., advisory boards) might look good at face value, but refuse to give community members the power needed to make change and offer consequential insight to the research process. In fact, advisory boards can cause more harm than good, furthering the divide between PWUD and researchers. With this considered, NCSU has installed processes, largely informed by the principles of community driven research (CDR) [7], to avoid such experiences. Research collaborations begin with an informal meeting between NCSU and researchers to discuss objectives, methods, findings dissemination, party responsibilities, and other logistics. Project ideas are born out of mutual collaboration, as opposed to researchers approaching the organization with a fully developed project in hand. Instead, the proposed framework encourages community-initiated research questions, centering the needs of those performing the behaviors in question. This meeting lays the groundwork for a mutually beneficial and respectful collaboration, while developing relevant research ideas aimed to directly benefit the community. As projects materialize, infrastructure is created to allow for expression of needed modifications and expectations. Collaborations have resulted in conducting on-site needs assessment surveys, in-person interviews, development of health education materials, and more. Following project implementation, dispersal of results prioritizes community-based utilization. NCSU, along with its research partners, ensures undertaken projects will be as beneficial for community members as they are for academics. 

Many other harm reduction programs like NCSU exist across the country. These organizations present a unique opportunity for collaborations between researchers and PWUD. In fact, researchers and PWUD should not be considered as two mutually exclusive roles. Rather, research institutions should make a concerted effort to hire PWUD and create opportunities for career growth. This may be sought through a number of potential paths, including collaboration with community-led organizations who can facilitate ongoing, mutually beneficial relationships between PWUD and research institutions, and emphasis on recruiting PWUD who are in pursuit of careers in research through traditional channels (i.e., higher education and formal research training) to join staff. However, it is important to note institutional barriers lock many PWUD out of conventional mechanisms of research training, and consequently long-term employment in the field of research. Obstructions are frequently due to prior felony convictions, as the war on drugs has pushed so many PWUD into encounters with the criminal justice system. As such, it is imperative to the growth of the field of substance use research that research institutions learn to better navigate the line between accountability and flexibility. As long as research institutions uphold these obstacles to the advancement of PWUD in the field of research, they amplify the influence of the criminalization of substance use and inhibit maximized benefit of research efforts on public health. Still, we caution researchers from simply hiring or collaborating with PWUD without thorough thought about how to establish and maintain a relationship grounded in respect and openness, in addition to holding a mission to improve the health and wellbeing of PWUD.

## Research as a career path for PWUD

Currently, the common career paths for PWUD accepted by society are limited to those serving other PWUD in social service settings, such as peer support specialists. While providing services to others can be beneficial to both the persons receiving and delivering the services, these jobs are often underpaid and can lack critical benefits that traditionally accompany other types of full-time employment (e.g., health insurance, retirement benefits) [8]. We argue research is another field in which employment of PWUD would be mutually beneficial. The field of research is filled with many accomplished individuals with respective areas of expertise. PWUD, too, hold their own area of expertise: drug use and the many related health factors and outcomes. Rather than stigmatize PWUD for their use, we should see them for their value as experts. Due to long held stigmatization of drug use, discrimination can present in the researcher themselves, their organizations, and the policies that fund research. Researchers may even suppress or withhold information about their own drug use in order to protect their professional reputation and employment status from drug-related stigma. Therefore, to change the landscape of drug use research, we argue it is the responsibility of researchers to enact change by creating employment opportunities for PWUD in which their expertise is valued and respected and where they are seen as equals with their colleagues [9].

To this end, the power imbalances between PWUD and formally trained researchers must be dismantled. While we would certainly encourage PWUD hoping to pursue research as a career path to follow continuing education and relevant certification opportunities, barriers to these formalized systems of education must be acknowledged. Furthermore, a credential or degree does not replace the invaluable experiential knowledge held by PWUD. As such, credentials and degrees should not serve as the standard by which we judge a person’s capacity to contribute to the research process. Rather, it is the responsibility of the researcher, and relevant partnering organizations, to empower PWUD to participate in the research process in a meaningful way. Dependent upon the research project, we recommend project and role specific training for PWUD, as well as equitable allocation of legitimate decision-making power. Likewise, it is essential researchers nurture long-lasting relationships with non-profits and other community-led organizations they intend to work with. As opposed to only interacting during the project period, which is not conducive to ongoing training for PWUD, enduring relationships foster continuing professional development as well as collaborations built in trust. These strategies will furnish PWUD with the skills and opportunities necessary to genuinely influence the research process.

To be clear: we are not suggesting researchers include any and all people who *have* used drugs. Instead, we are advocating for increasing the participation of PWUD who have the relevant experience and expertise with the drug types, consumption techniques, and environments being investigated. Particularly given the current fluidity of the drug market, those actively engaged are best situated to speak to present-day trends. Moreover, it is vital PWUD included in the research process possess the situational understanding to critically assess the current use environment and contribute to impactful investigation. These individuals are well positioned to provide accurate and timely information that can strengthen analyses of the drug, set, and setting factors that influence consumption behavior and negative health outcomes [10]. Drug user unions like NCSU are already playing an integral role in public health research by aiding researchers to better involve PWUD in research efforts. Concisely stated: something can be statistically meaningful when chasing *p*-values but be socially and medically irrelevant for effectiveness and successful implementation.

Representation and guidance from PWUD is also critical to translating scientific knowledge gained through research into improved public policies and direct service practices. Some public health authorities approach that translation process through advisory boards or other bodies with a nominal ‘voice’, but the inclusion of PWUD among bodies with a formal ‘vote’ remains rare. Oregon’s Drug Addiction Treatment and Recovery Act of 2020, passed by ballot initiative in November 2020, decriminalized possession of small amounts of controlled substances and redirected cannabis tax revenues to an Oversight and Accountability Council tasked with overseeing grants to implement Addiction Recovery Centers and increase access to community care [11]. The Oversight and Accountability Council is required to include at least two members, “who suffered or suffer from substance use disorder,” and while that language would be improved by centering on members’ lived experience without the characterization of ‘suffering’, the inclusion of people who use(d) drugs as voting members of a body empowered to direct public funding for drug-related health and social services provides an opportunity for increasingly equitable structures of public policy and governance.

## Conclusions

If the goal, especially during a lethal overdose epidemic, is to conduct research that can contribute to the development of useful, evidence-based interventions the likes of needle exchange, lay naloxone distribution, onsite wound care, safer injection education, fentanyl test strips, and peer navigation, then it is plainly obvious researchers need to include and expand efforts to collaborate with PWUD. Given the rising rates of drug-involved morbidity and mortality, it is high time to include people who use drugs in public health efforts. Our lives depend on it.

## Data Availability

Not applicable.

## Abbreviations

PWUD: People who use drugs
SSPs: Syringe service programs
NCSU: North Carolina Survivors Union
NIH: National Institutes of Health
FDA: U.S. Food and Drug Administration
CDR: Community driven research
